# Resuspended Nano-Minerals in Coal Ash: A Potential Factor in Elevated Lung Cancer Rates in Xuanwei and Fuyuan, Yunnan, China

**DOI:** 10.3390/toxics12120919

**Published:** 2024-12-19

**Authors:** Wenhua Wang, Mengyang Wang, Longyi Shao, Jiajia Shao, Pengju Liu

**Affiliations:** 1 School of Resources and Civil Engineering, Northeastern University, Shenyang 110819, China; wangwenhua@neuq.edu.cn (W.W.);; 2 School of Resources and Materials, Northeastern University at Qinhuangdao, Qinhuangdao 066004, China; 3 State Key Laboratory of Coal Resources and Safe Mining & College of Geosciences and Surveying Engineering, China University of Mining and Technology, Beijing 100083, China; pj-liu@mail.tsinghua.edu.cn; 4 State Environmental Protection Key Laboratory of Sources and Control of Air Pollution Complex, State Key Joint Laboratory of Environmental Simulation and Pollution Control, School of Environment, Tsinghua University, Beijing 100084, China

**Keywords:** Xuanwei, lung cancer, coal ash, quartz, Fe-rich particle

## Abstract

Xuanwei and the neighboring Fuyuan (XF) counties in Yunnan Province have the highest lung cancer incidence rates in China. Previous studies suggest that the nano-minerals released during the combustion of locally sourced “smoky” (bituminous) coal are the primary contributors to these elevated cancer rates. The coal ash generated during combustion predominantly consists of nano-minerals, which can be resuspended into the atmosphere during routine ash-handling activities. In this study, coal ash samples from XF counties and four additional provinces with lower lung cancer incidence rates were resuspended to simulate ash-handling activities and subsequently collected using a cascade PM_2.5_ sampler. Individual particles were analyzed using a high-resolution scanning electron microscope coupled with energy-dispersive X-ray spectroscopy (SEM-EDX). Based on their morphology and elemental composition, the particles were categorized into five major types: quartz, Si- and Al-rich (SiAl-rich), Ca-rich, Ca- and Mg-rich (CaMg-rich), and Fe-rich particles. The relative abundance of crystalline quartz particles was significantly higher in Xuanwei (22.2%) and Fuyuan (13.7%) compared to the other provinces, where quartz was also detected in lower concentrations. Similarly, the proportion of Fe-rich particles was notably higher in Xuanwei (10.9%) and Fuyuan (5.1%) than in other regions. These findings highlight the potential role of quartz and Fe-rich particles in contributing to the high lung cancer rates observed in XF counties. Further research is warranted to elucidate the toxicological mechanisms underlying the health effects of these particle types.

## 1. Introduction

Lung cancer is one of the most common malignant tumors and remains the leading cause of cancer-related deaths worldwide [1,2,3]. According to the World Health Organization, there were approximately 2.48 million new cases of lung cancer and 1.82 million lung cancer-related deaths in 2022 (https://gco.iarc.fr/today/home; accessed on 19 December 2024). Various risk factors, including smoking [4], air pollution [5,6], and genetic predispositions [7], have been reported to be highly associated with lung cancer.

Xuanwei and the neighboring Fuyuan (XF) counties, located in eastern Yunnan Province, exhibit some of the highest lung cancer incidence and mortality rates in China [8,9,10]. Epidemiological studies have revealed the unique characteristics of lung cancer in these regions [11]. First, the incidence rate is notably higher in rural areas compared to urban areas [1,11]. Furthermore, the lung cancer incidence rates among women in XF counties are comparable to those among men, with non-smoking women in these regions exhibiting the highest lung cancer incidence globally [1,12,13].

Coal resources are abundant in XF counties, where coal burning is widely used for household cooking and heating, particularly in rural areas [11]. The combustion of coal releases substantial amounts of particulate and gaseous pollutants, causing severe indoor air pollution that adversely impacts human health [14]. Previous research has closely linked the high lung cancer incidence in XF counties to the burning of local “smoky” (bituminous) coal [15,16,17], although genetic factors may also play a significant role [13].

To better understand the relationship between coal combustion emissions and lung cancer incidence in XF counties, the physicochemical properties of indoor air pollutants and particles emitted from experimental coal burning have been extensively analyzed [12,18,19,20,21]. Factors such as heavy metals [14,16] and indoor polycyclic aromatic hydrocarbons (PAHs) [11,20] have been identified as potential contributors to the high lung cancer rates. Notably, nano-sized quartz has been found in both the pulmonary and normal tissues of lung cancer patients [1,19]. Since 1997, crystalline quartz has been classified as a Group 1 carcinogen for humans by the International Agency for Research on Cancer (IARC) [22]. When inhaled, quartz particles can deposit deep within the lungs, triggering chronic inflammation and oxidative stress [23,24]. Moreover, quartz exposure can induce pulmonary fibrosis, further increasing cancer risk by promoting abnormal cell proliferation in lung tissue [25]. Therefore, the role of nano-sized quartz generated during coal combustion was highlighted as the primary driver of lung cancer [1,19]. However, Downward et al. [18] argued that indoor quartz concentrations were not particularly high in XF counties and proposed that most quartz particles are emitted as part of coal ash.

Coal ash, a significant by-product of coal burning, contains various minerals and can be easily resuspended into the air during daily cooking and ash-handling activities. During such activities, individuals are directly exposed to high particle loads, which, upon inhalation, pose severe health risks.

High-resolution scanning electron microscopy (SEM) is a powerful tool for characterizing the morphology and elemental composition of individual aerosol particles [15,26,27,28,29,30]. In this study, coal samples from XF counties in Yunnan Province and four other provinces were combusted. The resulting coal ash was resuspended to simulate ash-handling activities and subsequently collected using a cascade PM_2.5_ sampler. Individual ash particles were analyzed for their morphological and elemental composition using high-resolution SEM coupled with energy-dispersive X-ray spectroscopy (EDX).

## 2. Materials and Methods

### 2.1. Sample Collection

In this study, six types of coal were analyzed. Two coal samples were collected from Xuanwei and Fuyuan counties in Yunnan Province, regions with the highest lung cancer rates in rural China. Considering the significance of Shanxi Province, Shandong Province, and Inner Mongolia Autonomous Region as major coal-producing areas in China, three additional coal samples were obtained from Datong in Shanxi, Jining in Shandong, and Ordos in Inner Mongolia. The final coal sample was obtained from a rural household in Beijing. As its origin could not be determined, this sample was classified as having an unknown origin.

**Figure 1:**
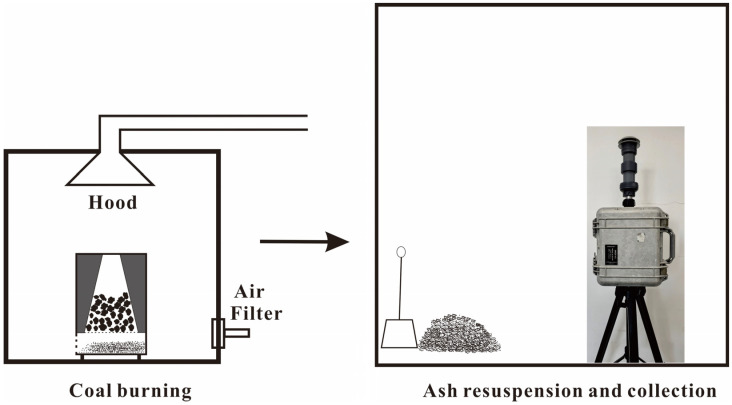
Diagram showing coal burning and ash collection system.

The coal samples were combusted in a laboratory combustion chamber, as shown in Figure 1. During combustion, most of the organic components were burned off, leaving behind coal ash residues that primarily accumulated at the bottom of the chamber. The collected bottom ash was transferred to a larger room, where it was manually stirred using a small spade to simulate real-word ash-handling activities. This stirring caused some of the ash to become resuspended in the air. The resuspended ash particles were collected on polycarbonate filters (Millipore, Feltham, UK) using a cascade PM_2.5_ sampler (MiniVolTAS, Eugene, OR, USA). The sampler was positioned approximately 1 m above ground level, representing the typical breathing height when individuals lower their heads during ash-handling activities. This setup was designed to closely simulate human exposure conditions during such scenarios.

Each type of coal was tested independently. To verify the reliability of the process, the coals from Xuanwei were tested twice. The results of the repeated experiments were consistent, as shown in Table S1.

### 2.2. SEM Observation

The morphology and elemental composition of individual coal ash particles were analyzed using an SEM (FEI Ltd., Hillsborough, FL, USA) in accordance with the National Standard of the People’s Republic of China (GB/T 35099-2018). This standardized approach ensures high accuracy and reproducibility in characterizing particulate matter. The experimental procedure involved the following steps:

(1) Sample Preparation: A section of approximately 1 cm^2^ was cut from each polycarbonate filter containing the collected ash particles. The cut filter segment was then mounted onto a sample holder using conductive carbon tape to secure the sample and ensure uniform contact.
(2) Coating Process: The mounted samples were coated with a thin layer of platinum (Pt) using a sputter coater. This coating step was essential to enhance the electrical conductivity of the samples, which mitigates charging effects under the electron beam. The Pt coating also improved the clarity and resolution of the SEM images, enabling more precise morphological analysis.
(3) SEM Analysis: The prepared samples were analyzed at an accelerating voltage of 20 kV. To ensure the representativeness of the analysis, several fields at low magnification were randomly selected in each sample, and all the particles within these fields were imaged at magnifications ranging from ×5000 to ×20,000. This range allowed for the identification of both overall particle morphology and fine structural details, facilitating a comprehensive analysis.

## 3. Results

### 3.1. Classification of Ash Particles

**Figure 2:**
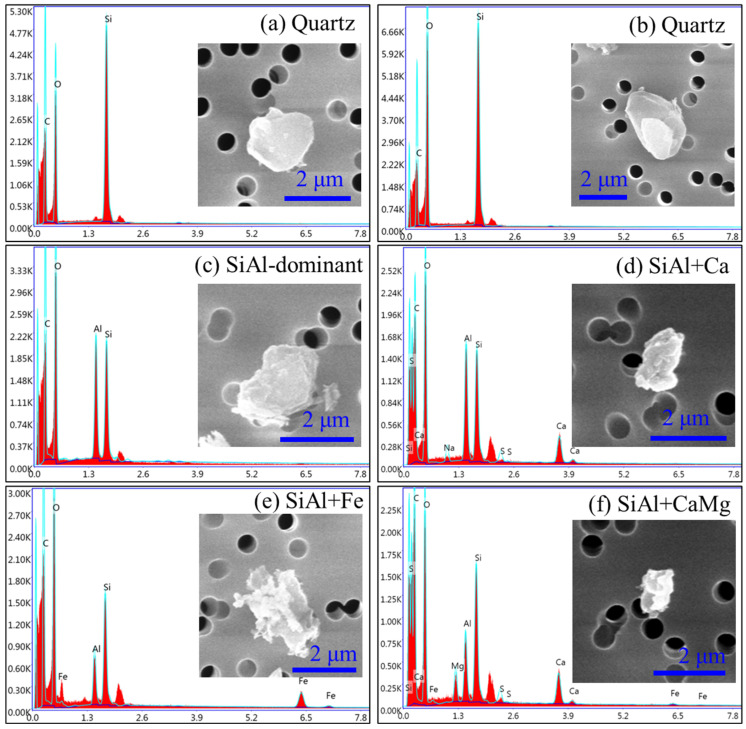
Morphology and elemental composition of quartz and SiAl-rich particles.

**Figure 3:**
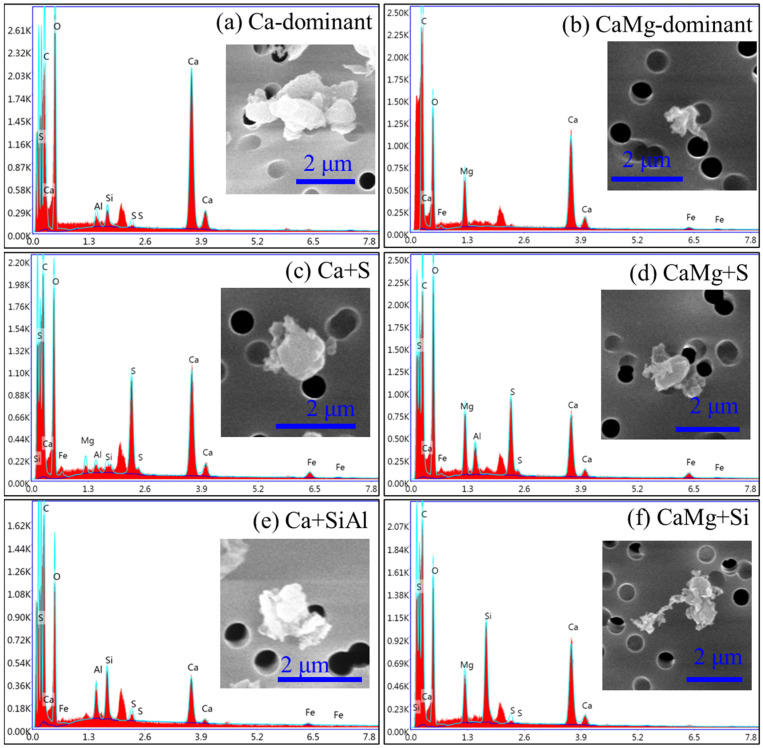
Morphology and elemental composition of Ca-rich and CaMg-rich particles.

**Figure 4:**
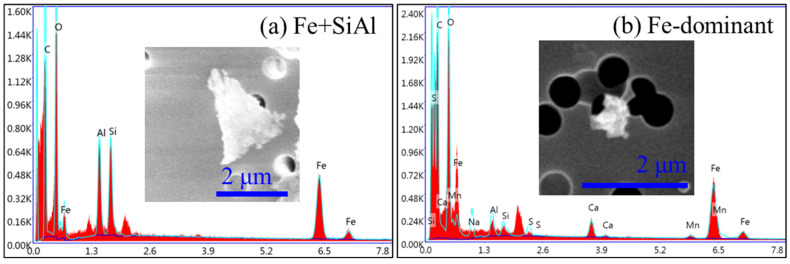
Morphology and elemental composition of Fe-rich particles.

Due to the low burning temperatures compared to those at a thermal power plant, the particles in this study exhibited irregular shapes. As such, ash particles were classified based on their main elemental composition (weight percentage rather than element counts) obtained from EDX. The particles were classified into five types: quartz, Si- and Al-rich (SiAl-rich), Ca-rich, Ca- and Mg-rich (CaMg-rich), and Fe-rich. The detailed morphology and elemental composition of each particle type are shown in Figure 2, Figure 3 and Figure 4.

If the particles were primarily composed of silicon (Si) and oxygen (O), as illustrated in Figure 2a,b, they were classified as quartz. These particles typically exhibited a smooth surface morphology, distinguishing them from other particle types. If the particles were dominated by O, Si, and Al, they were classified as SiAl-rich particles. These particles were aluminosilicates and also contained a small proportion of other elements. Therefore, they were further classified into “SiAl-dominant” (Figure 2c), “SiAl + Ca” (Figure 2d), “SiAl + Fe” (Figure 2e), and “SiAl + CaMg” (Figure 2f) subtype particles.

If the particles were mainly composed of Ca and O, they were classified as Ca-rich particles. If the particles were mainly composed of Ca, Mg, and O, they were classified as CaMg-rich particles. They can also have minor amounts of other elements, as shown in Figure 3. Therefore, they were further classified into different subtypes. “Ca-dominant” particles were mainly composed of Ca and O, as shown in Figure 3a, and they were calcite. Some other subtypes of Ca-rich particles, such as “Ca + S” (Figure 3c) and “Ca + Si/Al” (Figure 3e) subtype particles, were also found. “CaMg-dominant” particles were mainly composed of Ca, Mg, and O, as shown in Figure 3b, and they were dolomite. Some other subtypes of CaMg-rich particles, such as “CaMg + S” (Figure 3d) and “CaMg + Si/Al” (Figure 3f) subtype particles, were also found.

If the particles were dominated by Fe and O, they were classified as Fe-rich particles, which were further classified into “Fe-dominant” and “Fe + Si/Al” subtype particles. “Fe-dominant” particles were mainly composed of Fe and O with very small amounts of other elements, as shown in Figure 4b. “Fe + Si/Al” particles showed the highest weight ratios of Fe on individual ash particles and also had higher weight ratios of Si/Al, as shown in Figure 4a.

Some other particles such as Ti-rich particles, as shown in Figure S1, accounted for a small percentage, and they were classified into other particle types.

### 3.2. Relative Percentage of Ash Particles

The relative percentages of different particle types in coal ashes from various regions are summarized in Table 1. The coal-burning particles in Xuanwei county were predominantly composed of SiAl-rich particles (39.3%), followed by quartz (22.2%) and Ca-rich (25.5%) particles. In contrast, Fuyuan county’s coal ashes contained a higher proportion of SiAl-rich particles (67.5%) and a smaller percentage of quartz particles (13.7%). Notably, no CaMg-rich particles were detected in the coal ashes from Xuanwei and Fuyuan counties in this study.

Fe-rich particles were more abundant in Xuanwei (10.9%) and Fuyuan (5.1%) ashes compared to those from other regions, as shown in Table 1. In contrast, the coal ashes from Shanxi Province and the Inner Mongolia Autonomous Region primarily consisted of SiAl-rich particles (33.7% and 34.9%, respectively) and Ca-rich particles (40.2% and 31.0%, respectively), with CaMg-rich particles accounting for 14.1% and 26.7%, respectively.

Interestingly, coal ashes from Shandong Province and the unidentified source exhibited a high dominance of CaMg-rich particles, comprising 89.8% and 57.6% of the total particle number, respectively. Although the overall particle types were similar across some regions, significant variations were observed in the subtypes of coal ash particles. For instance, “Ca + S” and “CaMg + S” subtype particles were more prevalent in the coal ashes from the Inner Mongolia Autonomous Region, Shandong Province, and the unknown source compared to those from Shanxi Province. This variation in subtypes could be attributed to differences in coal composition.

## 4. Discussions and Health Implications

The elevated lung cancer incidence in XF counties of Yunnan Province can be attributed to multiple factors. While genetic predisposition has been highlighted by epidemiological studies as a contributing factor [13], extensive research has established a strong correlation between the high lung cancer rates and the burning of domestic “smoky” coal. Combustion-related components, such as PAHs [11,20], heavy metals [14,16], and nano-minerals [12], are considered the primary contributors.

This study compared coal ash particles from XF counties with those from other regions using SEM-EDX analysis. The results demonstrated that coal ash particles in these counties were predominantly irregular shaped and retained their crystalline structures. This is likely a consequence of the lower combustion temperatures in domestic stoves compared to thermal power plants, allowing the inorganic components to remain crystalline [31,32,33]. It has been reported that the irregular-shaped crystalline particles exhibited more toxicology than the amorphous ones [22,34].

A significant finding was the high proportion of quartz in the resuspended ash particles from XF counties, with quartz comprising 22.2% of the particles in Xuanwei and 13.7% in Fuyuan. Downward et al. [18] reported that indoor air quartz levels in these counties were not exceptionally high. Nano-quartz particles were found in the tissues of lung cancer patients in XF counties [19]. Our results highlighted that ash-handling activities might cause short-time high PM pollution, and resuspended quartz might play an important role in the elevated lung cancer rates.

Another notable finding was the higher prevalence of Fe-rich particles in XF coal ashes, accounting for 10.9% in Xuanwei and 5.1% in Fuyuan. The inhalation of Fe-rich particles has been associated with increased lung cancer risk due to their role in the Fenton reaction [8]. Upon inhalation, Fe-rich particles can deposit into the lungs and react with hydrogen peroxide (H_2_O_2_), which is naturally present in the body, to generate hydroxyl radicals (·OH) through the following reaction [35]:

Fe^2+^ + H_2_O_2_ → Fe^3+^ + ·OH + OH^−^ (1)

The generated hydroxyl radicals are highly reactive and are considered the most damaging reactive oxygen species (ROS), capable of causing extensive damage to cellular components, including lipids, proteins, and DNA [36]. Previous studies have shown that the rate of iron ion release depends on the mineralogy of iron. The iron oxides in clays can release iron ions more easily than iron oxides [8,37,38]. In this study, Fe-rich particles also contained small amounts of silicon (Si) and aluminum (Al), which could further exacerbate the health risk associated with their inhalation.

In conclusion, this research emphasizes that the high concentrations of quartz combined with Fe-rich particles in XF counties may contribute to the elevated lung cancer incidence in the region. The toxicological mechanisms underlying the health effects of these particles warrant further investigation.

## 5. Conclusions

Xuanwei and Fuyuan (XF) counties have the highest lung cancer rates in China. In this study, coal samples were collected from XF counties and four other provinces. The coals were combusted, and the resulting coal ashes were resuspended into the atmosphere to simulate ash-handling activities. The morphology and elemental composition of the resuspended particles were analyzed using high-resolution electron microscopy coupled with energy-dispersive X-ray spectroscopy.

The results highlight that the resuspended ash particles in XF counties contained significantly higher proportions of quartz, with 22.2% in Xuanwei and 13.7% in Fuyuan. Additionally, Fe-rich particles were also more prevalent in XF counties, accounting for 10.9% in Xuanwei and 5.1% in Fuyuan. Given the known toxicity of quartz and Fe-rich particles when inhaled, these findings highlight their potential role in the high lung cancer incidence observed in the region.

## 6. Outlook

This study is the first to identify higher proportions of quartz and Fe-rich particles in resuspended coal ash particles in XF counties through laboratory simulations, linking them to the high lung cancer rates in the regions. However, this work is constrained by the limited set and reliance on laboratory simulations. Therefore, we propose the following directions for future research: (1) expanding the sample set by collecting more coal samples from various locations; (2) conducting simulations at different heights to mimic both children and adults; (3) performing in situ sample collection in XF counties during ash-handling activities to compare with simulations; (4) conducting multiple toxicological experiments to explore the biological effects of inhaled quartz and Fe-rich particles in relation to the high lung cancer rates observed in these areas, and based on the above results (5) conducting health risk assessments to evaluate the potential impacts of exposure to these particles on public health in the region.

## Figures and Tables

**Table 1:** Relative percentage of different particle types from different coal ashes.

Particle Types	Xuanwei	Fuyuan	Shanxi	Neimeng	Shandong	Unknown
Quartz	22.2%	13.7%	7.3%	5.2%	0.9%	5.1%
SiAl-rich	39.3%	67.5%	33.7%	34.9%	1.0%	9.3%
SiAl-dominant	28.0%	59.0%	18.4%	13.4%	--	2.5%
SiAl + Ca	2.9%	2.6%	8.5%	17.7%	0.9%	5.1%
SiAl + CaMg	--	--	4.3%	3.9%	--	1.7%
SiAl + Fe	8.4%	6.0%	2.6%	--	--	--
Ca-rich	25.5%	8.5%	40.2%	31.0%	8.3%	28.0%
Ca-dominant	16.3%	3.4%	19.2%	0.4%	2.8%	5.1%
Ca + Si/Al	5.4%	1.7%	14.5%	6.9%	2.8%	9.3%
Ca + S	3.8%	3.4%	6.4%	23.7%	2.8%	13.6%
CaMg-rich	0.0%	0.0%	14.1%	26.7%	89.8%	57.6%
CaMg-dominant	--	--	4.3%	1.7%	53.7%	6.8%
CaMg + Si/Al	--	--	4.3%	14.7%	6.5%	5.1%
CaMg + S	--	--	5.6%	10.3%	29.6%	45.8%
Fe-rich	10.9%	5.1%	2.1%	1.3%	0.0%	0.0%
Fe-dominant	--	0.9%	0.4%	--	--	--
Fe + Si/Al	10.9%	4.3%	0.4%	1.3%	--	--
Fe + Ca	--	--	1.3%	--	--	--
Others	2.1%	5.1%	2.6%	0.9%	0.0%	0.0%

## Data Availability

Data are contained within this article and the Supplementary Materials.

## Supplementary Materials

The following supporting information can be downloaded at https://www.mdpi.com/article/10.3390/toxics12120919/s1, Figure S1: Morphology and elemental compositions of Ti-rich particles. Table S1: Relative number percentage of different particle types from two independent experiment.
