# Supplementary material for: Resuspended Nano-Minerals in Coal Ash: A Potential Factor in Elevated Lung Cancer Rates in Xuanwei and Fuyuan, Yunnan, China
Source: Toxics. 2024 Dec 19;12(12):919. doi: 10.3390/toxics12120919 (PMC11728513; doi:10.3390/toxics12120919)
Supplement: Supplementary file 1 [file toxics-12-00919-s001.zip › toxics-3351613-supplementary.pdf]

Supporting information for

Resuspended Nano-Minerals in Coal Ash: A Potential Factor in Elevated Lung Cancer Rates in Xuanwei and Fuyuan, Yunnan, China

Wenhua Wang[1,2], Mengyang Wang[1,2], Longyi Shao[3*], Jiajia Shao[1,2], Pengju Liu[3,4]

1 School of Resources and Civil Engineering, Northeastern University, Shenyang, 110819, China
2 School of Resources and Materials, Northeastern University at Qinhuangdao, Qinhuangdao, 066004, China
3. State Key Laboratory of Coal Resources and Safe Mining & College of Geosciences and Surveying Engineering, China University of Mining and Technology, Beijing, 100083, China
4. State Environmental Protection Key Laboratory of Sources and Control of Air Pollution Complex, State Key Joint Laboratory of Environmental Simulation and Pollution Control, School of Environment, Tsinghua University, Beijing, 100084, China
* Correspondence: ShaoL@cumtb.edu.cn

Table S1. Relative number percentage of different particle types from two independent experiment.

Particle Types	Replicate Experiment 1	Replicate Experiment 1	Average
**Quartz**	**25.0%**	**19.3%**	**22.2%**
**SiAl-rich**	**38.3%**	**40.3%**	**39.3%**
SiAl-dominant	26.7%	29.4%	28.0%
SiAl+Ca	3.3%	2.5%	2.9%
SiAl+CaMg	--	--	--
SiAl+Fe	8.3%	8.4%	8.4%
**Ca-rich**	**23.3%**	**27.7%**	**25.5%**
Ca-dominant	13.3%	19.3%	16.3%
Ca+Si/Al	6.7%	4.2%	5.4%
Ca+S	3.3%	4.2%	3.8%
**CaMg-rich**	**0.0%**	**0.0%**	**0.0%**
CaMg-dominant	--	--	--
CaMg+Si/Al	--	--	--
CaMg+S	--	--	--
**Fe-rich**	**10.8%**	**10.9%**	**10.9%**
Fe-dominant	--	--	--
Fe+Si/Al	10.8%	10.9%	10.9%
Fe+Ca	--	--	--
**Others**	**2.5%**	**1.7%**	**2.1%**

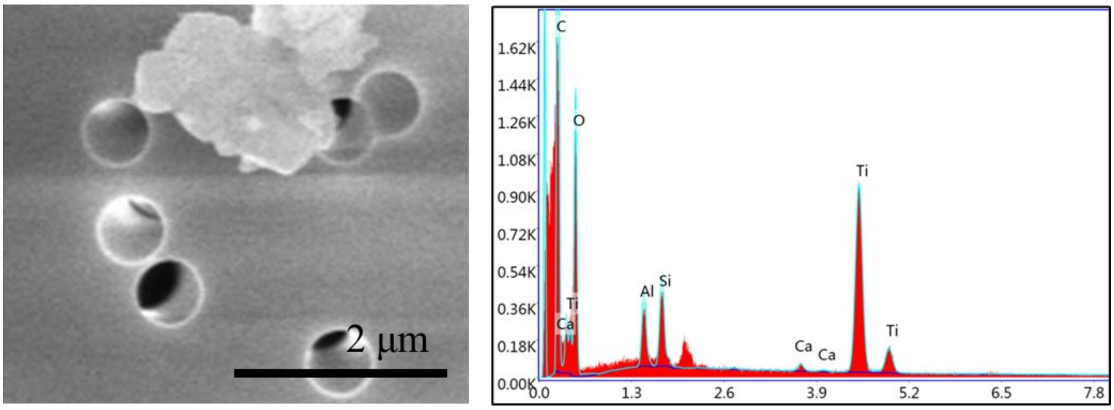

Fig. S1 Morphology and elemental composition of Ti-rich particle.
